# Atlas of Pathological Histology

**Published:** 1853-05

**Authors:** 


Art. IX.—*Atlas of Pathological Histology.* By Dr. Gottlieb Gluge, Professor of Physiology and Pathological Anatomy in the University of Bruxelles, Member of the Royal Academy of Bruxelles, &c. Translated from the German by Joseph Leidy, M. D., Pathologist to St. Joseph's Hospital, Philadelphia, Fellow of the College of Physicians, Philadelphia, Honorary Fellow of the Medical Society of Virginia, Corresponding Member of the Biological Society of Paris, &c., &c.

"No apology," says the Translator in his preface, "can be necessary for presenting to the Medical profession in the United States, the translation of a work upon a subject, relatively so new to the Science of Medicine as Pathological Histology. Its importance to Pathological Anatomy, is of the same character as normal histology is to normal anatomy, and this cannot be better represented than by referring to the great and permanent advance which Physiology has made in its relation to the physical structure of the organs of the living body. Pathological Anatomy also is beyond doubt of the highest value in medicine, for a scientific treatment of disease must, of necessity, depend to a very considerable extent upon our knowledge of material changes which are so frequently the source of those symptoms which indicate its existence. The present volume of Gluge, originally appended to his great work on Pathological Anatomy, besides illustrating the various subjects of pathological histology, accompanied by copious references, will be found of particular interest from the light which is thrown upon the inflammatory process, and its various attendant and accessory phenomena."

We are very sure that such of our brethren as are interested in the matters treated of in this work, will not only agree with the Translator that "no apology" is "necessary for presenting it to the medical profession in the United States," but will join us in heartily thanking him for doing so.—It is such men as the Author who add most to the *Science* of Medicine.

Patient, careful and laborious in observing, faithful, precise and minute in recording, the results of their toil always instruct, always profit.

We are aware of the objections sometimes proclaimed aloud—oftener murmured lowly or vaguely insinuated against the practical utility of microscopic investigations in the study of morbid and cadaveric phenomena. We are aware too of the proclivity of many minds continually to exalt the *new* at the expense of the *old*, without duly weighing and carefully collating and comparing each—and to find in each successive track of medical investigation flowery paths and verdant fields, and purling brooks and leafy shades, without thinking once of poisonous berries and venomous reptiles and hidden and deceitful enchantments, and profitless if not dangerous wanderings.

In the pursuit of no other science can there be found so many progressionists and so many ultra conservatists, as in the science of Medicine. Multitudes of its devotees are the worst kind of old Fogies—multitudes are no less blind, perverse and obstinate in their enthusiasm for progression. The former will not voluntarily allow science to make a solitary advance; the latter will have her go over a precipice rather than not go at all. The former, unwilling themselves to push on, sneer at and deride the tottering steps of those who try to walk under the heavy burden—the latter thinking to help, in their officious, inconsiderate and misdirected enthusiasm, do but stumble and fall, and sometimes drag down the abler, steadier carriers with them.

Something of the spirit which animates this latter class, has repeatedly exhibited itself in reference to this very subject:—indeed, there are those who place more reliance upon the revelations of the microscope than upon any other thing whatsoever. Such, it seems to us, are no better, if not worse, than those who see neither beauty nor knowledge nor profit, so far as medical science and art are concerned, in all the pictures painted on the field of every instrument in existence. These but oppose a progress which is irresistible—but sneer at facts which can bear sneering—slight discoveries which can bear slighting—deride realities which can laugh derision to scorn, and reject truths, the rejection of which involves loss mainly to the unbelievers. Those, on the contrary, in thinking to add to the temple of science, build walls which the winds of scrutiny overthrow, rear spires which the lightnings of intellect strike to the earth, and add roofs which the fire of reason utterly consumes—and lo! is left, not a grander, more beautiful and more magnificent edifice, but the same, blackened a little by the fire, and encumbered with heaps of rubbish.

To leave metaphors, we think the *real value* of this kind of investigation has been much underrated by many, and the *results* much overrated by a few:—the latter by those who expect to find herein the elements of diseases—the proximate cause and real essence: the former who forget or have never taken the trouble to inform themselves of what has really been done in this way, and now look forward hopefully to the full development through the separate steps of progress, all imperfect and some unreal.

It is certainly true that Physiology has been a very great gainer by microscopic researches. It is equally true, we think that Pathology as yet has not. While we admit, with the Translator, that "pathological anatomy is beyond doubt of the highest value in medicine," we are constrained to deny the implication that any "great and permanent advance" has been made in our pathological knowledge by the use of the microscope. We have been told many things about many curious sights, to be seen by bringing into the field discovered particles, but all that we have been told, has not as yet added one iota to the real value of our medical knowledge. And while we look forward with an assured belief to the evolution of something true and tangible from the chaotic mass of discoveries, real and assumed, hereafter to be and heretofore presented to our notice, we never expect that from this source Pathology and Pathological Anatomy will so much benefit as Physiology and Normal Anatomy has done and will do.

This expectation is founded in the fact that the difficulties which beset the study of the latter, are, in one respect at least, different in kind and greater in degree than those which embarrass the former, viz. in this—the more varying nature and relations of diseased than of healthy elements.

Nevertheless something really has been done—surely something more will be. And in this belief and this confident expectation, we are glad that this most interesting work has been made accessible to us. Interesting it is to every body, and important to all who are engaged in conducting such researches—or who seek information on this subject from reliable sources.

The Volume contains text and plates.—

The text is divided into seven sections, treating of *Development of the elements of tissues*, in which are elaborately discussed, all formations and the development of fibres. 2nd. *The elements of the tissues combined in perfect and imperfect tissues and arranged according to the processes of disease*, in which are considered the two classes of tissues imperfectly developed—cytoblastema, nucleoli, nuclei and celles: the transition forms to perfect tissues and those so perfected. 3d. *Formation of Blastema*, embracing nutrition, secretion: Inflammation and its terminations: Congestion; Hyperannia and Stasis, their causes: Pus in general, its formation,—the diagnosis of pus corpuscles: the chemical relation and its varieties;—and Granulation and Cicatrization. 4th. *The histological metamorphosis of*

*the blood*, within the arteries, veins and heart and exterior bloodvessels. 5th. *Pyæmia*, under which are treated, the conditions of Pyæmia, the purulent dyscrasia with "observations" on each. 6th. *Gangrene.* 7th. *Observations on Histology*, relative to Stasis and exudation, Calcification, Atheroma, Cysto Sarcoma, Colloid Cysto-Steatoma, Albuminous Sarcoma, Cancer—its transition forms and degeneration, Enteritis, Typhoid fever, scarlatina and Glanders.

The plates are twelve in number—the aggregate figures amounting to more than three hundred. The first plate contains forty seven of these, illustrating the transformation of the blood, exudation, corpuscles and pus. The second, thirty eight, of Granulation, Hyperæmia and Exudation. The third illustrating Steatosis, twenty. The fourth, Ossification, twenty six. The fifth, Cells, fibres and cell-fibres in tumors, forty two. The sixth, Formation of fibres and cells in Cancer, nineteen. The seventh, formation of cells, Cancer and Pigment, twenty five. The eigth, Intestinal glands and Epithelia in Typhus and Scarlatina, seventeen. The ninth, Glands and Epithelia in Cholera, twenty six. The tenth, eleventh, and twelfth, Entozoa, Epizoa, Epithelia, &c., fifty four.

We are glad to be able to commend, and that in somewhat emphatic terms, the mechanical execution, both print and plates of this work. The latter are far better than the common run of plates as found in our medical books—plates of which the best that can be said usually, is, that they are wholly unlike what they are supposed to represent, as well as to the last degree coarse and slovenly as works of art. These are not so.

The volume begins with an introductory chapter containing five tables "of the magnitude and weight of the organs of man in the normal and abnormal condititions," with remarks and "observations"—all of very great interest—to us, more than any other part of the work, which as a whole we especially commend to our readers.

